# Association of childhood meat consumption with late-life cognitive function: mediation by adult education and moderation by parental literacy

**DOI:** 10.3389/fpubh.2026.1878281

**Published:** 2026-10-05

**Authors:** Zhihao Chai, Rao Dai, Baijing Zhou, Chaoqiang Jiang, Yali Jin, Feng Zhu, Tai Hing Lam, Lin Xu, Weisen Zhang

**Affiliations:** 1 Guangzhou Twelfth People's Hospital, Guangzhou, China; 2 Greater Bay Area Public Health Research Collaboration, Guangzhou, China; 3 School of Public Health, Guangzhou Medical University, Guangzhou, China; 4 School of Public Health, Sun Yat-sen University, Guangzhou, China; 5 School of Public Health, The University of Hong Kong, Hong Kong, China; 6 Institute of Applied Health Research, University of Birmingham, Birmingham, United Kingdom

**Keywords:** adult education, childhood meat consumption, cognition, mediation, moderation, parental literacy

## Abstract

**Background:**

Results on the associations of childhood nutrition with late-life cognitive function are inconsistent, and little is known about whether family educational resources modify this association or whether adult education lies on the life-course pathway. We examined the associations of childhood meat consumption (CMC), used as an indicator of childhood nutrition, with late-life cognitive function and mild cognitive impairment (MCI). Moreover, we further examined whether these associations varied by sex, age, parental literacy, and socioeconomic status, and whether they were mediated by adult education.

**Methods:**

Guangzhou Biobank Cohort Study (GBCS) participants, who were 50 years old and above with Guangzhou household registration and recruited from 2006 to 2008 were included. CMC was reported as never/about once a year, about once a month, about once a week, or almost daily. Cognitive function was assessed using the Mini-Mental State Examination (MMSE), its four domains, and the Delayed 10-Word Recall Test (DWRT). Information on parental literacy and adult education was also collected. Multivariate linear, logistic regression and mediation analyses were done.

**Results:**

In 8,865 participants aged ≥50 years, after adjustment for sex, age, and parental literacy, compared with participants who ate meat never or about once a year during childhood, those who ate meat about once a month, once a week and almost daily had higher MMSE scores [β (95% *CI*): 0.33 (0.17–0.49), 0.83 (0.68–0.99) and 1.13 (0.93–1.32)] and lower odds of MCI [*OR* (95% *CI*): 0.84 (0.71–1.00), 0.55 (0.45–0.66) and 0.31 (0.22–0.42)], respectively. Associations were strongest for orientation. Frequent CMC (≥1/week) was more strongly associated with MMSE score [β (95% *CI*): 0.73 (0.63–0.84)] among participants with literate parents and lower odds of MCI [*OR* (95% *CI*): 0.45 (0.38–0.54)] than among those with illiterate parents. Adult education mediated 40% [95% *CI*: 34.1%−47.3%] of the association between CMC and MMSE. Consistent results were found using DWRT.

**Conclusion:**

More frequent CMC was associated with better late-life cognitive function and lower MCI prevalence, particularly among participants with literate parents. Adult education accounted for a substantial proportion of the association. These results provide new evidence that improving early-life nutrition and promoting educational attainment during early and mid-life may contribute to delaying and preventing cognitive impairment in later adulthood.

## 1 Introduction

Cognitive function is typically assessed using the Mini-Mental State Examination (MMSE) scale as a screening tool. Mild cognitive impairment (MCI) is an intermediate stage between normal cognitive aging and dementia, characterized by a noticeable decline in memory or other cognitive abilities that does not yet interfere significantly with daily activities (1). In 2022, the global prevalence of MCI in community-dwelling populations exceeded 15% (2), consistent with findings from a large cross-sectional study of 46,011 Chinese adults in 2018, which reported a prevalence of 15.5% (3). Previous studies have shown that approximately 10%−15% of individuals with MCI progress to dementia annually, with the conversion rate reaching up to 80% over 6 years, compared with only 1%−2% of cognitively normal older adults (4). Given the substantially elevated risk of progression to dementia among individuals with MCI, identifying modifiable factors across the life course represents an important public health priority.

Nutrition during childhood supports brain growth, myelination, neurotransmitter synthesis, and the development of cognitive abilities. Most studies on early life nutrition have focused on children or adolescents, generally showing a positive association with cognitive outcomes (5–7). Research in older adults has relied mainly on indicators such as arm length, knee height, leg length, self-reported hunger, or famine exposure. Some studies found that better early-life nutritional conditions were associated with better cognitive performance or lower cognitive impairment. A cross-sectional study of Chinese aged 65–105 showed that early-life nutritional deprivation, using proxies such as arm length, knee height and self-reported hunger, was associated with cognitive impairment (8). Furthermore, a cross-sectional study conducted in a Chinese population showed that individuals exposed to famine during childhood had a higher risk of MCI than those unexposed (9). Importantly, Heys et al. (10) reported that more frequent childhood meat eating was associated with better immediate and delayed word recall and with amnesic MCI. That study focused primarily on memory-related outcomes and treated education and socioeconomic position as covariates; it did not examine MMSE global and domain-specific cognition, parental literacy as an effect modifier, or statistical mediation by adult education and other adulthood socioeconomic factors. These questions therefore remain unresolved.

However, other studies reported null or age- and sex-specific associations. A cross-sectional study involving middle-aged and older Chinese adults showed that knee height was associated with memory-related cognitive function in individuals aged 60 and above, but the coefficient became statistically insignificant in those aged 45–59 (11). A cross-sectional study of urban older adults in Latin America and the Caribbean showed no association between physical health, earlier-life hunger, and adult cognition function (12). These associations may vary across the different historical contexts, as individuals from developing countries, particularly older adults in developing countries may have experienced more severe and prolonged nutritional deprivation in early life (11). It remains uncertain whether early-life nutrition is consistently associated with late-life cognitive function and whether such associations, if present, are contingent upon parental education.

During the early years of the People's Republic of China, many older Chinese adults experienced hardships during childhood, including World War II and the Great Famine of 1959–1961 (13). In the context of widespread food scarcity and limited access to formal education, childhood meat consumption (CMC) reflected children's access to energy, high-quality protein, micronutrients, vitamins, and other animal-source nutrients (14), whereas parental literacy may indicate early educational exposure and family educational resources. Parental literacy may also shape cognitive aging by providing greater cognitive stimulation, facilitating school participation, and helping translate early nutritional advantages into sustained educational opportunities (15). Educational attainment in adulthood may subsequently contribute to cognitive reserve, occupational complexity, income, health literacy, and healthier aging (16). However, it remains unclear whether the association between CMC and late-life cognition differs across cognitive domains, whether it is modified by parental literacy, and which adulthood pathways may partly account for this association.

We therefore examined the associations of CMC with the total MMSE score, four MMSE domains (orientation, memory, attention and calculation, recall and language), and the Delayed 10-Word Recall Test (DWRT) score, and MCI among middle-aged and older Chinese adults, using data from the Guangzhou Biobank Cohort Study (GBCS). We hypothesized that more frequent CMC would be associated with better cognitive outcomes and lower MCI prevalence. Notably, a meta-analysis of 47 studies involving 137,599 Chinese older adults found that the prevalence of MCI in Chinese older adults decreases with the increase of education level: the prevalence of MCI in the illiterate group (years of education <1 year) is 30% [95% *CI* (25%−35%)], while that in the university group (years of education >12 years) drops to 10% [95% *CI* (7%−13%)] (17). Based on this, we further hypothesized that these associations would vary by parental literacy and other sociodemographic factors, and that adult education and related socioeconomic factors would partly mediate these associations.

## 2 Materials and methods

### 2.1 Study participants

All participants were recruited from the Guangzhou Biobank Cohort Study (GBCS). Briefly, GBCS is a three-way collaborative project among the Guangzhou Twelfth People's Hospital, the University of Hong Kong, China, and Birmingham, UK. GBCS is an ongoing population-based prospective cohort study including relatively healthy individuals aged 50 years or above. Participants were recruited from the Guangzhou Health and Happiness Association for the Respectable Elders (GHHARE), a large social welfare organization covering approximately 7% of the older population in Guangzhou, with branches in all districts of Guangzhou (18). The baseline data for phases 1 to 3 of the GBCS were collected from September 2003 to January 2008. In phase 3 (2006–2008) and all subsequent follow-up phases, we additionally used the MMSE to measure cognitive function. Hence, the analysis in the present study only included 10,088 participants recruited at the baseline of the phase 3.

Of 10,088 phase-3 participants, 1,223 were excluded due to missing data of CMC and/or parental literacy (755), MMSE and/or DWRT (322), and model covariates (146). A total of 8,865 participants (2,221 men and 6,644 women) with complete data on all variables of interest were included in the present analysis. The participant flow is shown in Figure 1. Data were collected in a face-to-face interview using a computerized questionnaire. The questionnaire's test-retest reliability was evaluated through repeat interviews in 200 randomly selected participants approximately 1 month later and was considered satisfactory (18). The Guangzhou Medical Ethics Committee of the Chinese Medical Association approved the study, and all participants provided written informed consent.

**Figure 1:**
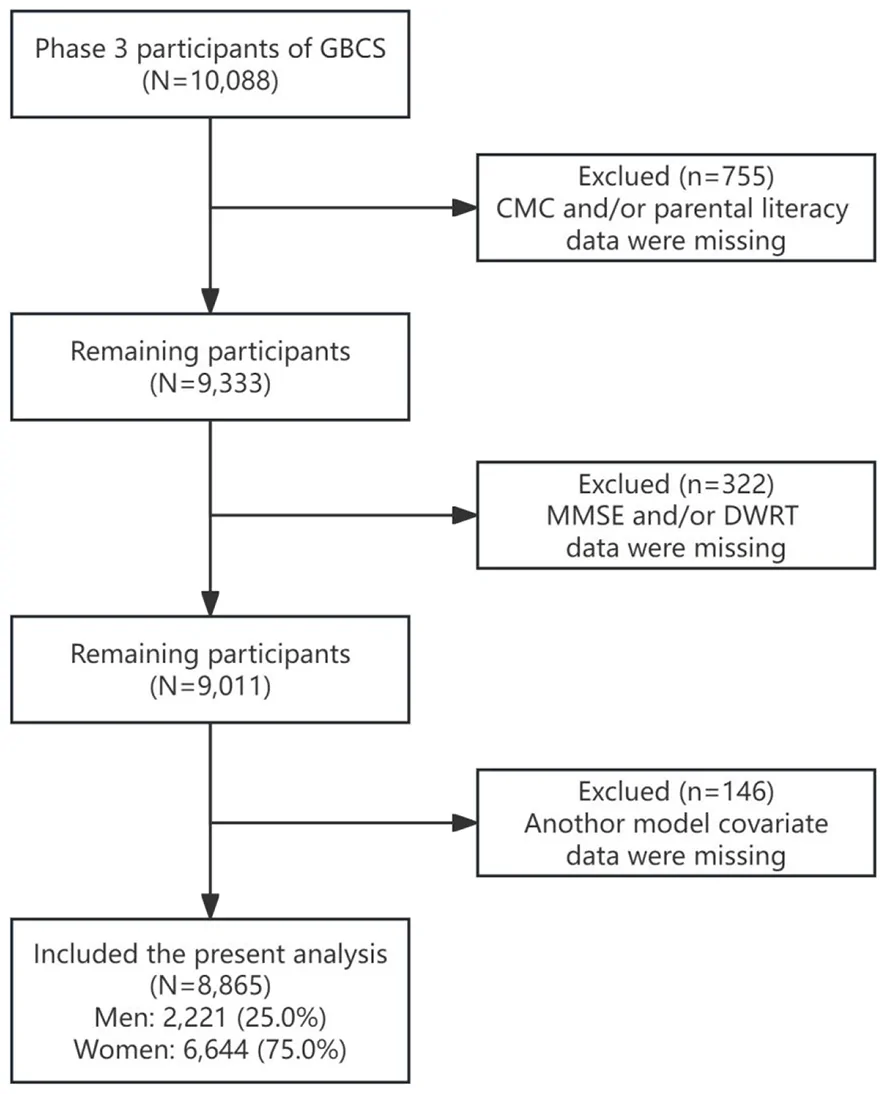
Participant flow diagram. GBCS, Guangzhou Biobank Cohort Study; MMSE, mini-mental state examination; DWRT, delayed 10-word recall test.

### 2.2 Childhood meat consumption

CMC was used as a proxy for childhood dietary and nutritional conditions (10), rather than as a comprehensive measure of childhood nutritional status. Information on CMC was obtained by asking, “How often did you eat meat as a child?” Response options were never, about once a year, about once a month, about once a week, and almost daily. Because fewer than 1% reported never eating meat, “never” and “about once a year” were combined. The four categories: never or about once a year (0 points), about once a month (1 point), about once a week (2 points), and almost daily (3 points). The CMC score ranged from 0 to 3, with higher scores indicating higher frequency of consumption. The questionnaire captured frequency only and did not assess portion size, type of meat, total protein intake, other animal- or plant-based protein sources, or overall dietary quality. For interaction analyses, participants were further classified into two categories as in frequent CMC (CMC score <2) and frequent CMC (CMC score ≥2) based on the median CMC score of 2.

### 2.3 Cognitive outcomes

The outcomes were cognitive function and MCI, assessed by MMSE and DWRT. Details using MMSE/DWRT were reported in our previous GBCS paper (19–22). In brief, MMSE, developed by Folstein et al. (65), is a widely used 30-point test to measure cognitive function, which is recognized as the preferred scale for dementia screening and is included in the diagnostic examination outline for the U.S. ECA psychiatric epidemiology survey and the composite international diagnostic examination recommended by WHO. It covers four domains: orientation (10 points), memory (immediate and short-term memory, 6 points), attention and calculation (5 points), recall and language (9 points), with total scores ranging from 0 to 30. Higher scores indicate better cognitive function. Scores from 1 to 24 indicate cognitive impairment.

The DWRT is a test on verbal learning and recent memory, which requires recalling a list of words after a 5-min delay. First, 10 simple Chinese words (soy sauce, arm, letter, chairman, ticket, grass, corner, stone, book and stick) were read out to the participants one by one, after which the participants were immediately instructed to recall the words. This procedure was repeated 3 times. Following a 5-min delay after the third repetition, participants were asked to recall the 10 words once more. The DWRT score was the number of correct words recalled after the 5-min delay, ranging from 0 to 10, with higher scores indicating better short-term memory function of cognition. This assessment method is consistent with the memory testing mode in professional cognitive assessment tools like the Montreal Cognitive Assessment (MoCA), where examiners read words at a rate of one per second and ask subjects to recall them after a period of time, and it also aligns with the common short-term memory evaluation methods in clinical cognitive function assessment. MCI was defined by MMSE scores <25 or DWRT scores <5 (23).

### 2.4 Potential confounders, effect modifiers and mediators

In model 1, sex, age (in years), and parental literacy were prespecified as core adjustment variables. Parental literacy was categorized into two groups: neither parent could read/write, and at least one parent could read/write. They were also included as potential confounders and effect modifiers. Socioeconomic position, lifestyle factors, and health status were included as mediators in model 2. The socioeconomic factors included marital status (married or unmarried or others), adult education (primary school and below, middle school, or college and above), family annual income (<10,000, 10,000–29,999, 30,000–49,999, ≥50,000 CYN/year, or not know, US$1 = 7 CNY), and occupation (manual workers, non-manual workers, or others). Lifestyle factors included smoking, drinking, and physical activity. The status of smoking and drinking was categorized as “never, former, and current.” Physical activity was assessed using the International Physical Activity Questionnaire (IPAQ), which was validated in our previous study, and was divided into three groups: inactive, moderate and active (24, 25). The health status factors included body mass index (BMI) (continuous variable), self-rated health status, cardiovascular disease, diabetes, and depressive symptoms. BMI was calculated as weight in kilograms divided by body height in meters squared (kg/m^2^). Depressive symptoms were assessed using a previously validated version of the 15-item Geriatric Depression Scale (GDS-15) (26). The presence of depressive symptoms was defined as a GDS-15 score of 5 or higher as in our previous GBCS paper (27, 28).

### 2.5 Statistical analysis

Continuous variables were presented as means (standard deviation), and categorical variables as numbers (percentages, %). Analysis of variance (ANOVA) was employed to compare the characteristics of continuous variables, while the chi-square test was used for categorical variables. All domains were converted into *Z* scores to facilitate the comparison of the associations between CMC and cognitive functions across the four domains. Multivariable linear regression was used to analyze the association of CMC with MMSE (total and each of the four domains) and DWRT scores. Because residual diagnostics suggested possible heteroscedasticity, a sensitivity analysis was performed using a generalized linear model with a normal distribution, an identity link function, and a robust covariance estimator based on the Huber–White sandwich method. Logistic regression was used to analyze the association of CMC with MCI, giving adjusted regression coefficients (βs), odds ratios (*OR*s) and 95% confidence intervals (*CI*s).

Previous studies have identified age and educational attainment as important factors associated with cognitive outcomes in older adults and individuals with MCI (29). Based on these findings and our *a priori* hypotheses, potential effect modification by sex, age, parental literacy, and socioeconomic status (SES) was examined by including the corresponding interaction terms in the regression models, followed by stratified analyses when statistically significant interactions were identified. We used mediation analysis to estimate the contribution of potential mediators to the association of CMC scores with MMSE and DWRT scores. For mediation analyses, potential mediators were dichotomized, i.e., adult education (primary or below vs. middle school or above), family income (<30,000 CNY/year vs. ≥30,000 CNY/year), occupation (non-manual vs. manual or others), marital status (married vs. unmarried or others), physical activity (moderate or above vs. inactive), smoking status (never vs. ever), drinking status (never vs. ever), self-rated health (good vs. poor), cardiovascular (no vs. yes), diabetes (no vs. yes), and depressive (no vs. yes) in adulthood.

Model 1 was adjusted for sex, age, and parental literacy. Model 2 additionally adjusted for adult socioeconomic position, marital status, lifestyle, body mass index, self-rated health, cardiovascular disease, diabetes, and depressive symptoms. Model 1 was used as a minimally adjusted model. Covariates in Model 2 were selected *a priori* according to a life-course framework and previous literature, rather than by stepwise selection or by retaining variables based on statistical significance.

The “mediation” package in *R* was used to perform mediation analysis. All tests were two-sided, and a *P* value <0.05 was considered statistical significance. All statistical analyses were performed using IBM SPSS Statistics 25.0 and R software.

## 3 Results

### 3.1 Participant characteristics

Table 1 shows that participants with CMC never or about once a year were included more men, older people, single individuals, more physically active, and never smokers, but fewer current drinkers (all *P* < 0.05). They had lower SES (lower adult education, lower family annual income, and more manual occupations). They also had lower BMI, poorer self-rated health, more depressive symptoms, and more illiterate parents (all *P* < 0.01).

**Table 1:** Characteristics by childhood meat consumption in 8,865 participants in Guangzhou Biobank Cohort Study.

Variables	All	Childhood meat consumption				*P-*value	*P* for trend
		Never or about once a year	About once a month	About once a week	Almost daily		
**Number of participants (%)**	8,865 (100.0)	1,752 (19.8)	2,649 (29.9)	3,306 (37.3)	1,158 (13.1)		
**Age, years, mean (SD)**	59.8 (7.7)	63.0 (7.9)	60.1 (7.6)	58.3 (7.0)	58.7 (7.7)	<0.001	
Age group, years, *n* (%)							
<60	5,017 (56.6)	684 (39.0)	1,464 (55.3)	2,138 (64.7)	731 (63.1)	<0.001	
≥60	3,848 (43.4)	1,068 (61.0)	1,185 (44.7)	1,168 (35.3)	427 (36.9)		
Sex, *n* (%)							
Women	6,644 (74.9)	1,314 (75.0)	1,934 (73.0)	2,504 (75.7)	892 (77.0)	0.03	
Men	2,221 (25.1)	438 (25.0)	715 (27.0)	802 (24.3)	266 (23.0)		
Adult education, *n* (%)							
≤ Primary	3,187 (36.0)	1,044 (59.6)	1,126 (42.5)	786 (23.8)	231 (19.9)	<0.001	<0.001
Middle school	4,901 (55.3)	611 (34.9)	1,358 (51.3)	2,171 (65.7)	761 (65.7)		
≥College	7,77 (8.7)	97 (5.5)	165 (6.2)	349 (10.5)	166 (14.4)		
Family income, CNY/year, *n* (%)							
<10,000	444 (5.0)	167 (9.6)	160 (6.1)	102 (3.1)	15 (1.3)	<0.001	<0.001
10,000–29,999	2,698 (30.4)	578 (33.0)	869 (32.8)	956 (28.9)	295 (25.5)		
30,000–4,9999	2,293 (25.9)	342 (19.5)	686 (25.9)	949 (28.7)	316 (27.3)		
≥50,000	2,029 (22.9)	249 (14.2)	494 (18.6)	898 (27.2)	388 (33.5)		
Not know	1,401 (15.8)	416 (23.7)	440 (16.6)	401 (12.1)	144 (12.4)		
Occupation, *n* (%)							
Manual	5,401 (60.9)	1,233 (70.4)	1,714 (64.7)	1,877 (56.8)	577 (49.8)	<0.001	0.008
Non-manual	1,833 (20.7)	257 (14.6)	481 (18.2)	779 (23.6)	316 (27.3)		
Others	1,631 (18.4)	262 (15.0)	454 (17.1)	650 (19.6)	265 (22.9)		
Marital status, *n* (%)							
Married	7,391 (83.4)	1,371 (78.3)	2,207 (83.3)	2,847 (86.1)	966 (83.4)	<0.001	
Unmarried or others	1,474 (16.6)	381 (21.7)	442 (16.7)	459 (13.9)	192 (16.6)		
Physical activity, *n* (%)							
Inactive	928 (10.5)	157 (9.0)	327 (12.4)	343 (10.4)	101 (8.7)	0.002	0.98
Moderate	2,522 (28.4)	486 (27.7)	740 (27.9)	972 (29.4)	324 (28.0)		
Active	5,415 (61.1)	1,109 (63.3)	1,582 (59.7)	1,991 (60.2)	733 (63.3)		
Smoking status, *n* (%)							
Never	7,325 (82.6)	1,413 (80.7)	2,138 (80.7)	2,796 (84.6)	978 (84.5)	<0.001	0.002
Former	669 (7.6)	166 (9.4)	231 (8.7)	194 (5.8)	78 (6.7)		
Current	871 (9.8)	173 (9.9)	280 (10.6)	316 (9.6)	102 (8.8)		
Drinking status, *n* (%)							
Never	4,495 (50.7)	958 (54.7)	1,362 (51.4)	1,622 (49.1)	553 (47.8)	<0.001	<0.001
Former	171 (1.9)	58 (3.3)	45 (1.7)	45 (1.3)	23 (1.9)		
Current	4,199 (47.4)	736 (42.0)	1,242 (46.9)	1,639 (49.6)	582 (50.3)		
**BMI, kg/m** ^**2**^ **, mean (SD)**	23.8 (3.3)	23.9 (3.5)	24.0 (3.4)	23.7 (3.3)	24.0 (3.2)	0.001	
**Self-rated health, poor**, ***n*** **(%)**	1,740 (20.8)	391 (23.7)	573 (22.7)	600 (19.6)	176 (15.9)	<0.001	
**Cardiovascular disease, yes**, ***n*** **(%)**	3,576 (40.3)	719 (41.0)	1,068 (40.3)	1,296 (39.2)	493 (42.6)	0.21	
**Diabetes, yes**, ***n*** **(%)**	636 (7.2)	139 (7.9)	184 (6.9)	228 (6.9)	85 (7.3)	0.54	
**Depressive symptoms, yes**, ***n*** **(%)**	1,261 (14.2)	330 (18.8)	407 (15.4)	392 (11.9)	132 (11.4)	<0.001	
Parental literacy, *n* (%)							
Neither parent could read/write	1,478 (16.7)	598 (34.1)	522 (19.7)	282 (8.5)	76 (6.6)	<0.001	
At least one parent could read/write	7,387 (83.3)	1,154 (65.9)	2,127 (80.3)	3,024 (91.5)	1,082 (93.4)		

SD, standard deviation; CNY, Chinese Yuan; BMI, body mass index; MMSE, mini-mental state examination; DWRT, delayed 10-word recall test; MCI, mild cognitive impairment.

### 3.2 CMC and overall cognitive function

Table 2 shows that in Model 1, after adjusting for sex, age, and parental literacy, compared with those with CMC never or about once a year, those with CMC about once a month, about once a week and almost daily showed higher MMSE scores, with β (95% *CI*) of 0.33 (0.17–0.49), 0.83 (0.68–0.99) and 1.13 (0.93–1.32), respectively. Additionally, these participants had lower odds of MCI (defined as MMSE <25), with adds ratios (*OR*s, 95% confidence intervals [*CI*s]) of 0.84 (0.71–1.00), 0.55 (0.45–0.66) and 0.31 (0.22–0.42), respectively. Model 2 showed that, after further adjusting for all covariates, the associations remained significant, although some associations were attenuated. Significant dose-response relationships were found (all *P* for trend <0.01). Consistent results were observed for MCI defined by DWRT scores (<5).

**Table 2:** Associations of childhood meat consumption with late adulthood cognitive function in 8,865 participants in Guangzhou Biobank Cohort Study.

Childhood meat consumption	Mean (SD)/*N* (%)	Crude model	Model 1	Model 2
Outcome = MMSE scores (0–30), β (95% *CI*)				
Never or about once a year	26.3 (3.6)	Ref (0.00)	Ref (0.00)	Ref (0.00)
About once a month	27.1 (2.9)	0.85 (0.68–1.01)^***^	0.33 (0.17–0.49)^***^	0.14 (−0.01–0.29)
About once a week	28.0 (2.2)	1.68 (1.53–1.84)^***^	0.83 (0.68–0.99)^***^	0.33 (0.18–0.48)^***^
Almost daily	28.2 (2.0)	1.94 (1.74–2.14)^***^	1.13 (0.93–1.32)^***^	0.52 (0.33–0.71)^***^
*P* for trend		<0.001	<0.001	<0.001
Outcome = DWRT scores (0–10), β (95% *CI*)				
Never or about once a year	5.5 (2.1)	Ref (0.00)	Ref (0.00)	Ref (0.00)
About once a month	5.9 (2.1)	0.43 (0.31–0.55)^***^	0.18 (0.06–0.29)^**^	0.12 (0.01–0.24)^*^
About once a week	6.3 (1.9)	0.81 (0.70–0.93)^***^	0.37 (0.25–0.48)^***^	0.18 (0.06–0.30)^**^
Almost daily	6.4 (1.9)	0.89 (0.75–1.04)^***^	0.46 (0.31–0.60)^***^	0.18 (0.03–0.33)^*^
*P* for trend		<0.001	<0.001	0.020
Outcome = MCI (MMSE scores<25), *OR* (95% *CI*)				
Never or about once a year	408 (23.3)	Ref (1.00)	Ref (1.00)	Ref (1.00)
About once a month	394 (14.9)	0.58 (0.49–0.68)^***^	0.84 (0.71–1.00)^*^	0.97 (0.81–1.16)
About once a week	258 (7.8)	0.28 (0.24–0.34)^***^	0.55 (0.45–0.66)^***^	0.84 (0.68–1.03)
Almost daily	59 (5.0)	0.17 (0.13–0.23)^***^	0.31 (0.22–0.42)^***^	0.56 (0.41–0.78)^**^
*P* for trend		<0.001	<0.001	0.003
Outcome = MCI (DWRT scores<5), *OR* (95% *CI*)				
Never or about once a year	525 (30.0)	Ref (1.00)	Ref (1.00)	Ref (1.00)
About once a month	582 (22.0)	0.66 (0.57–0.75)^***^	0.83 (0.72–0.96)^*^	0.87 (0.75–1.01)
About once a week	513 (15.5)	0.42 (0.36–0.48)^***^	0.64 (0.55–0.75)^***^	0.77 (0.66–0.91)^**^
Almost daily	178 (15.5)	0.40 (0.33–0.49)^***^	0.61 (0.49–0.74)^***^	0.82 (0.66–1.01)
*P* for trend		<0.001	<0.001	0.019

Ref, reference; CI, confidence interval; OR, odds ratio; MMSE, mini-mental state examination; DWRT, delayed 10-word recall test; MCI, mild cognitive impairment.

Model 1: adjusted for sex, age, and parental literacy.

Model 2: additionally adjusted for adult education, family annual income, occupation, marital status, physical activity, BMI, smoking status, drinking status, self-rated health, cardiovascular disease, diabetes and depressive symptoms.

^*^P < 0.05; ^**^P < 0.01; ^***^P < 0.001.

Diagnostic assessments indicated some deviation from normality and possible heteroscedasticity in residual distributions. Among the 8,865 participants, 131 (1.5%) were identified as potential outliers and 59 (0.7%) as high-leverage points. However, no observation showed excessive influence based on Cook's distance. Sensitivity analyses using heteroscedasticity-robust standard errors yielded estimates consistent with the primary analyses. The regression coefficients for participants with CMC occurring about once a month, about once a week and almost daily were 0.15 (−0.03 to 0.32), 0.36 (0.20 to 0.52), and 0.50 (0.32 to 0.68), respectively (Supplementary Tables 1, 2, and Supplementary Figure 1).

### 3.3 Cognitive domains

Table 3 shows that increasing CMC frequency was associated with higher scores in all four MMSE domains of orientation, memory, attention and calculation, recall and language, after adjusting for sex, age, and parental literacy in Model 1, and after adjusting for all covariates in Model 2 (*P* for trend from 0.01 to <0.001). Specifically, we found that the association between CMC and orientation domain was stronger than memory, attention and calculation, recall and language. Compared to those with CMC never or about once a year, those with CMC almost daily had higher orientation, memory, attention and calculation, recall and language, with standardized β (95% *CI*) of 0.18 (0.10–0.25), 0.05 (−0.03 to 0.12), 0.15 (0.08–0.22), and 0.11 (0.04–0.18), respectively.

**Table 3:** Associations of childhood meat consumption with present four domains scores of MMSE in 8,865 participants in Guangzhou Biobank Cohort Study.

Childhood meat consumption	Crude model	Model 1	Model 2
Orientation^a^, β (95% *CI*)			
Never or about once a year	Ref (0.00)	Ref (0.00)	Ref (0.00)
About once a month	0.29 (0.23–0.35)^***^	0.15 (0.09–0.21)^***^	0.09 (0.04–0.15)^**^
About once a week	0.50 (0.44–0.56)^***^	0.28 (0.22–0.33)^***^	0.14 (0.09–0.20)^***^
Almost daily	0.56 (0.49–0.63)^***^	0.34 (0.27–0.42)^***^	0.18 (0.10–0.25)^***^
*P* for trend	<0.001	<0.001	<0.001
Memory^a^, β (95% *CI*)			
Never or about once a year	Ref (0.00)	Ref (0.00)	Ref (0.00)
About once a month	0.14 (0.08–0.20)^***^	0.03 (−0.03–0.09)	−0.04 (−0.06–0.06)
About once a week	0.35 (0.30–0.41)^***^	0.17 (0.11–0.23)^***^	0.08 (0.02–0.14)^**^
Almost daily	0.35 (0.27–0.42)^***^	0.17 (0.10–0.24)^***^	0.05 (−0.03–0.12)
*P* for trend	<0.001	<0.001	0.014
Attention and calculation^a^, β (95% *CI*)			
Never or about once a year	Ref (0.00)	Ref (0.00)	Ref (0.00)
About once a month	0.19 (0.14–0.25)^***^	0.06 (0.01–0.12)^*^	0.02 (−0.04–0.07)
About once a week	0.38 (0.33–0.44)^***^	0.17 (0.12–0.23)^***^	0.05 (−0.01–0.11)
Almost daily	0.51 (0.44–0.58)^***^	0.31 (0.23–0.38)^***^	0.15 (0.08–0.22)^***^
*P* for trend	<0.001	<0.001	<0.001
Recall and language^a^, β (95% *CI*)			
Never or about once a year	Ref (0.00)	Ref (0.00)	Ref (0.00)
About once a month	0.23 (0.17–0.29)^***^	0.09 (0.03–0.15)^**^	0.03 (−0.03–0.09)
About once a week	0.45 (0.39–0.51)^***^	0.22 (0.17–0.28)^***^	0.08 (0.03–0.14)^**^
Almost daily	0.51 (0.43–0.58)^***^	0.29 (0.21–0.36)^***^	0.11 (0.04–0.18)^**^
*P* for trend	<0.001	<0.001	0.001

Ref, reference; CI, confidence interval; MMSE, mini-mental state examination.

^a^All domains were standardized using Z-scores to facilitate comparison.

Model 1: adjusted for sex, age, and parental literacy.

Model 2: additionally adjusted for adult education, family annual income, occupation, marital status, physical activity, BMI, smoking status, drinking status, self-rated health, cardiovascular disease, diabetes and depressive symptoms.

^*^P < 0.05; ^**^P < 0.01; ^***^P < 0.001.

### 3.4 Effect modification by parental literacy

We examined the potential moderation effects of sex, age, parental literacy, and SES on the relationship between CMC and late-life cognitive function. Of these factors, only parental literacy was found to exert a moderating effect on the aforementioned associations. The associations between CMC and both MMSE scores and having MCI (MMSE <25) varied by childhood parental literacy in Model 1 (both *P* for interaction <0.001). Subgroup analyses by parental literacy showed that, compared with participants whose parents were illiterate (β = 0.48, 95% *CI*: 0.06–0.89), the association between CMC and MMSE scores was stronger in those whose parents were literate (β = 0.73, 95% *CI*: 0.63–0.84). Participants whose parents were literate showed a significant association of CMC with lower odds of MCI (*OR* = 0.45, 95% *CI*: 0.38–0.54), but those whose parents were illiterate showed no significant association (*OR* = 0.87, 95% *CI*: 0.65–1.17). After adjusting for all covariates (Model 2), the significant effect moderation remained (*P* for interaction = 0.001 and 0.005, respectively), a higher frequency of CMC (≥1/week) was associated with higher MMSE scores (β = 0.35, 95% *CI*: 0.24–0.45) and lower odds of MCI (*OR* = 0.69, 95% *CI*: 0.57–0.83) in participants whose parents were literate. In contrast, no significant association was found between higher CMC frequency and MMSE scores or having MCI risk in participants whose parents were illiterate (Table 4).

**Table 4:** Associations of childhood meat consumption with present cognitive function in 8,865 participants in Guangzhou Biobank Cohort Study by parental literacy.

Childhood meat consumption	Crude model	Model 1	Model 2	Crude model	Model 1	Model 2
	Neither parent could read/write			At least one parent could read/write		
Outcome = MMSE scores (0–30), β (95% *CI*)						
<1/week	Ref (0.00)	Ref (0.00)	Ref (0.00)	Ref (0.00)	Ref (0.00)	Ref (0.00)
≥1/week	1.01 (0.57–1.45)^***^	0.48 (0.06–0.89)^*^	0.02 (−0.38–0.42)	0.95 (0.83–1.06)^***^	0.73 (0.63–0.84)^***^	0.35 (0.24–0.45)^***^
*P* for interaction				<0.001	<0.001	0.001
Outcome = DWRT scores (0–10), β (95% *CI*)						
<1/week	Ref (0.00)	Ref (0.00)	Ref (0.00)	Ref (0.00)	Ref (0.00)	Ref (0.00)
≥1/week	0.32 (0.07–0.57)^*^	0.15 (−0.10–0.39)	−0.03 (−0.28–0.22)	0.45 (0.36–0.53)^***^	0.30 (0.21–0.39)^***^	0.13 (0.04–0.22)^**^
*P* for interaction				<0.001	<0.001	0.057
Outcome = MCI (MMSE scores<25), *OR* (95% *CI*)						
<1/week	Ref (1.00)	Ref (1.00)	Ref (1.00)	Ref (1.00)	Ref (1.00)	Ref (1.00)
≥1/week	0.68 (0.52–0.90)^**^	0.87 (0.65–1.17)	1.12 (0.81–1.56)	0.38 (0.32–0.45)^***^	0.45 (0.38–0.54)^***^	0.69 (0.57–0.83)^***^
*P* for interaction				<0.001	<0.001	0.005
Outcome = MCI (DWRT scores<5), *OR* (95% *CI*)						
<1/week	Ref (1.00)	Ref (1.00)	Ref (1.00)	Ref (1.00)	Ref (1.00)	Ref (1.00)
≥1/week	0.74 (0.57–0.97)^*^	0.85 (0.64–1.12)	0.97 (0.72–1.29)	0.59 (0.52–0.67)^***^	0.68 (0.60–0.77)^***^	0.82 (0.72–0.94)^**^
*P* for interaction				0.12	0.15	0.28

Ref, reference; CI, confidence interval; OR, odds ratio; MMSE, mini-mental state examination; DWRT, delayed 10-word recall test; MCI, mild cognitive impairment.

Model 1: adjusted for sex, and age.

Model 2: additionally adjusted for adult education, family annual income, occupation, marital status, physical activity, BMI, smoking status, drinking status, self-rated health, cardiovascular disease, diabetes and depressive symptoms.

^*^P < 0.05; ^**^P < 0.01; ^***^P < 0.001.

### 3.5 Mediation

Table 5 shows that the association of CMC scores with MMSE scores was partly and mainly mediated by adult education, family income and occupation after adjusting for sex, age, and parental literacy, and the proportion of mediation was 40.00% (95% *CI*: 34.09%−47.26%), 10.92% (95% *CI*: 8.16%−14.28%) and 7.86% (95% *CI*: 5.80%−10.31%), respectively (all *P* < 0.001). Other minor mediators were smoking status, drinking status, self-rated health and depressive symptoms with mediation proportion, being 0.65% (95% *CI*: 0.03%−1.45%), 1.03% (95% *CI*: 0.06%−2.15%), 2.45% (95% *CI*: 1.20%−3.95%), and 4.92% (95% *CI*: 2.99%−7.30%), respectively (all *P* < 0.05). However, marital status, physical activity, BMI, cardiovascular disease and diabetes showed no mediation. Consistent results were found in the associations of CMC scores with DWRT scores.

**Table 5:** Associations of childhood meat consumption with present MMSE and DWRT scores with mediation by potential mediators.

Mediators	Indirect effect (ACME) Estimate (95% *CI*)^a^	Direct effect (ADE) Estimate (95% *CI*)^a^	Total effect Estimate (95% *CI*)^a^	Proportion by mediation % (95% *CI*)^a^
MMSE scores				
Adult education (middle school or above vs. primary or below)	0.1603 (0.1404–0.1814)^***^	0.2403 (0.1859–0.2944)^***^	0.4006 (0.3433–0.4575)^***^	40.00 (34.09–47.26)^***^
Family income^b^ (≥30,000 CNY/year vs. <30,000 CNY/year)	0.0416 (0.0314– 0.0526)^***^	0.3394 (0.2770–0.3990)^***^	0.3810 (0.3205– 0.4409)^***^	10.92 (8.16–14.28)^***^
Occupation (non-manual vs. manual or others)	0.0315 (0.0236–0.0402)^***^	0.3691 (0.3117–0.4257)^***^	0.4006 (0.3433–0.4575)^***^	7.86 (5.80– 10.31)^***^
Marital status (married vs. unmarried or others)	−0.0011 (−0.0042–0.0018)	0.4017 (0.3453– 0.4585)^***^	0.4006 (0.3433–0.4575)^***^	−0.28 (−1.10–0.43)
Physical activity (moderate or above vs. inactive)	0.0006 (−0.0019–0.0034)	0.4000 (0.3421– 0.4570)^***^	0.4006 (0.3433–0.4575)^***^	0.16 (−0.47–0.85)
Smoking (ever vs. never)	0.0026 (0.0001–0.0058)^*^	0.3980 (0.3403– 0.4554)^***^	0.4006 (0.3433–0.4575)^***^	0.65 (0.03–1.45)^*^
Alcohol drinking (ever vs. never)	0.0041 (0.0002–0.0086)^*^	0.3965 (0.3283– 0.4495)^***^	0.4006 (0.3433–0.4575)^***^	1.03 (0.06–2.15)^*^
BMI	−0.00004 (−0.0012–0.0010)	0.4006 (0.3433–0.4581)^***^	0.4006 (0.3433– 0.4575)^***^	−0.01 (−0.30–0.25)
Self-rated health (poor vs. good)	0.0098 (0.0048–0.0156)^***^	0.3908 (0.3336– 0.4479)^***^	0.4006 (0.3433–0.4575)^***^	2.45 (1.20– 3.95)^***^
Cardiovascular disease (yes vs. no)	0.0039 (−0.0003–0.0087)	0.3967 (0.3395– 0.4542)^***^	0.4006 (0.3433–0.4575)^***^	0.98 (−0.08–2.19)
Diabetes (yes vs. no)	0.0001 (−0.0007–0.0011)	0.4005 (0.3433–0.4575)^***^	0.4006 (0.3433–0.4575)^***^	0.02 (−0.19–0.28)
Depressive symptoms (yes vs. no)	0.0197 (0.0122– 0.0283)^***^	0.3809 (0.3228–0.4375)^***^	0.4006 (0.3433– 0.4575)^***^	4.92 (2.99–7.30)^***^
DWRT scores				
Adult education (middle school or above vs. primary or below)	0.0791 (0.0670– 0.0921)^***^	0.0828 (0.0406–0.1278)^***^	0.1619 (0.1197– 0.2074)^***^	48.85 (37.06–67.19)^***^
Family income^b^ (≥30,000 CNY/year vs. <30,000 CNY/year)	0.0253 (0.0181– 0.0332)^***^	0.1440 (0.0966–0.1910)^***^	0.1693 (0.1222– 0.2179)^***^	14.92 (10.04–22.26)^***^
Occupation (non-manual vs. manual or others)	0.0168 (0.0115–0.0223)^***^	0.1452 (0.1034–0.1907)^***^	0.1619 (0.1197–0.2074)^***^	10.36 (6.71– 15.46)^***^
Marital status (married vs. unmarried or others)	−0.0004 (−0.0018–0.0006)	0.1623 (0.1201– 0.2077)^***^	0.1619 (0.1197–0.2074)^***^	−0.24 (−1.20–0.40)
Physical activity (moderate or above vs. inactive)	0.0009 (−0.0026–0.0043)	0.1610 (0.1190– 0.2061)^***^	0.1619 (0.1197–0.2074)^***^	0.56 (−1.67–2.71)
Smoking (ever vs. never)	0.0012 (0.0001–0.0029)^*^	0.1608 (0.1134– 0.2050)^***^	0.1619 (0.1197–0.2074)^***^	0.73 (0.03–2.01)^*^
Alcohol drinking (ever vs. never)	0.0044 (0.0002–0.0092)^*^	0.1576 (0.1099– 0.2031)^***^	0.1619 (0.1197–0.2074)^***^	2.70 (0.13–5.93)^*^
BMI	0.00002 (−0.0006–0.0006)	0.1619 (0.1197–0.2072)^***^	0.1619 (0.1197– 0.2074)^***^	0.01 (−0.37–0.41)
Self-rated health (poor vs. good)	0.0053 (0.0024–0.0088)^***^	0.1567 (0.1141– 0.2012)^***^	0.1619 (0.1197–0.2074)^***^	3.26 (1.50– 5.80)^***^
Cardiovascular disease (yes vs. no)	0.0019 (−0.0002–0.0046)	0.1600 (0.1178– 0.2052)^***^	0.1619 (0.1197–0.2074)^***^	1.19 (−0.11–2.97)
Diabetes (yes vs. no)	−0.00004 (−0.0007–0.0005)	0.1620 (0.1146–0.2058)^***^	0.1619 (0.1197–0.2074)^***^	−0.03 (−0.46–0.31)
Depressive symptoms (yes vs. no)	0.0112 (0.0068–0.0163)^***^	0.1507 (0.1089– 0.1958)^***^	0.1619 (0.1197–0.2074)^***^	6.92 (4.06– 11.03)^***^

ACME, average causal mediated effect; ADE, average direct effect; *CI*, confidence interval; MMSE, mini-mental state examination; DWRT, delayed 10-word recall test; BMI, body mass index.

^a^Adjusting for sex, age, and parental literacy.

^b^Sample size = 7,464.

^*^P < 0.05; ^***^P < 0.001.

## 4 Discussion

In this large sample of middle-aged and older adults in Guangzhou, we extend previous GBCS work by examining several questions not addressed in the earlier analysis. Specifically, we assessed the association of CMC with global cognitive function and four MMSE domains, examined whether these associations differed according to parental literacy, and quantified the extent to which adult education and other adulthood socioeconomic factors statistically accounted for the observed associations. We additionally evaluated MCI using both MMSE- and DWRT-based definitions.

We first found that more frequent meat consumption during childhood was associated with better performance on two cognitive measures and lower prevalence of cognitive impairment. However, it should be noted that a study from Harvard University has warned that regular consumption of pork, beef and mutton in adulthood may increase the risk of dementia and accelerate cognitive aging, suggesting that the association between meat consumption and cognitive function may vary by life stage (30). The association between CMC and late-life cognitive function was also modified by parental literacy, being stronger among participants with literate parents and attenuated to non-significance among those with illiterate parents. Previous evidence linking parental education and involvement to better cognitive development in children (31, 32) provides contextual support for the possibility that family educational resources may help translate early nutritional advantages into longer-term cognitive benefits. Furthermore, we found that adult education was the main mediator, accounting for 40% of the associations between CMC and cognitive function on the MMSE and DWRT.

Our findings are consistent with previous studies using anthropometric indicators as proxies for early-life nutritional status. Several studies have suggested that better physical growth in early life is associated with better cognitive outcomes across the life course. For example, an analysis of four prospective birth cohorts found that greater early-life stature was associated with higher adult IQ (33). Similarly, in a prospective cohort of 12,368 term-born Belarusian children, greater length/height growth during the first 6.5 years of life was associated with higher cognitive scores at both 6.5 and 16 years of age (34). Evidence from older populations is also broadly consistent with these findings. Using data from the China Health and Retirement Longitudinal Study (CHARLS), Zhang et al. (11) found that greater knee height, a marker reflecting early-life growth and nutritional conditions, was associated with better episodic memory among Chinese adults aged ≥60 years. Our previous GBCS study similarly showed that greater adult height, sitting height, and leg length, used as indicators of childhood nutritional conditions, were associated with higher delayed recall scores (35). In addition, data from the Cardiovascular Health Cognition Study showed that greater knee height and arm span were associated with a lower risk of dementia (36).

Self-reported nutrition-related events were also used as indicators of early life nutrition. Zhang et al. (37) using data from the 2002 and 2005 waves of the Chinese Longitudinal Healthy Longevity Survey (CLHLS), showed that going to bed hungry during childhood was associated with a higher risk of cognitive impairment in old age. Similarly, Rong et al. (9) also demonstrated relevant associations based on their research. Moreover, Zhang et al. (38) using data from a retrospective birth cohort study, showed that daily milk consumption during childhood was associated with better late-life cognition, independent of other life course factors (38). Together, these findings support the possibility that more favorable nutritional and developmental conditions in early life may have long-term implications for cognitive health.

However, some studies reported conflicting findings. For example, Zheng et al. (39) using baseline data from the Health and Retirement Study (HRS), showed that after adjusting for socioeconomic status (SES), health behaviors, activity limitations, and diseases, higher adult height did not predict higher memory function at age 54. A cross-sectional study from South Korea reported no association between height and late-life cognitive function after adjusting for sociodemographic characteristics (40). Zhang et al. using data from CHARLS showed that in individuals aged 45–59 years, knee height was not significantly associated with episodic memory function after adjusting for childhood conditions, adulthood SES and health (11). Other studies have suggested that associations between indicators of early-life nutritional conditions and cognitive outcomes may differ by sex. Measures such as adult head circumference (41), knee height (36), leg length (37, 42), and a history of frequent hunger (37) have been associated with cognitive decline or impairment in women, but not consistently in men. These heterogeneous findings may reflect differences in study populations and birth cohorts, the timing and severity of early-life deprivation, exposure assessment, survival and selection processes, cognitive measures, and the extent of adjustment for adulthood socioeconomic and health-related factors.

Compared with anthropometric proxies of early-life nutritional conditions, CMC provides a more specific indicator of childhood dietary exposure and may therefore offer additional insight into potentially modifiable early-life factors. Importantly, our mediation analyses suggested that the association between CMC and late-life cognitive function may partly operate through socioeconomic pathways in adulthood. Adult education emerged as the strongest potential mediator, accounting for approximately 40% of the association with MMSE score, while family income and occupation showed smaller mediating proportions; several lifestyle and health-related factors contributed only modestly. These findings are broadly consistent with previous studies suggesting that adult socioeconomic circumstances may partly link childhood conditions to cognitive health in later life (43, 44). One possible interpretation is that better childhood nutritional conditions may facilitate educational attainment, which in turn may provide greater cognitive stimulation, occupational and socioeconomic opportunities, and cognitive reserve across the life course. Thus, the observed association between childhood nutrition and late-life cognitive function may reflect not only early nutritional advantages themselves, but also the accumulation of subsequent educational and socioeconomic advantages. From a public health perspective, these findings highlight the potential importance of improving childhood nutrition and promoting equitable access to education as complementary life-course strategies for supporting cognitive health in later life.

The “cognitive reserve hypothesis” may explain the observed associations, suggesting that early-life adversities, such as poverty, malnutrition, and lack of early parental education, can negatively influence brain development during childhood and adolescence (45, 46). Animal-source foods, including meat, provide high-quality protein and micronutrients such as iron, vitamin B12, zinc, and choline that are important for neurodevelopment, myelination, neurotransmitter synthesis, and synaptic function. Dietary protein supplies amino acids required for the synthesis of neurotransmitters such as serotonin, dopamine, and norepinephrine (47), while choline contributes to acetylcholine synthesis, cell membrane integrity, and brain development (48). Iron and vitamin B12 are also essential for normal neurological development and function, and deficiencies during sensitive periods of early life may have lasting adverse effects on cognitive development (49, 50). Experimental evidence further suggests that micronutrient deficiencies may disrupt hippocampal development and synaptic plasticity; for example, zinc deficiency has been shown to alter hippocampal synaptic ultrastructure and reduce synaptic vesicle numbers (51). Taken together, these findings provide biological plausibility for the possibility that inadequate nutrition during critical periods of brain development may have long-term consequences for cognitive health.

A complementary pathway may operate through the development of cognitive reserve across the life course. Parental literacy may provide greater cognitive stimulation, support school entry and persistence, improve health knowledge, and facilitate access to educational and social resources (15). These early advantages may promote higher educational attainment, which in turn may contribute to greater cognitive reserve and help maintain cognitive function in later life. Consistent with this possibility, lower educational attainment has been associated with more rapid hippocampal volume decline (52), while education-related cognitive enrichment has been linked to structural and functional brain characteristics that may support resilience to age-related cognitive decline (16, 53).

Taken together, these biological, educational, and social pathways suggest that early-life conditions may contribute to cognitive aging through cumulative processes across the life course. Childhood disadvantage may limit subsequent educational attainment, income potential, and occupational opportunities, thereby initiating chains of disadvantage that can persist into adulthood and influence later-life cognitive health (54, 55). Consistent with this life-course perspective, previous studies have linked adverse childhood circumstances to poorer cognitive function or faster cognitive decline in middle and older age (44, 46). These associations may operate partly through adult educational and socioeconomic circumstances, as well as psychosocial pathways such as depressive symptoms and social isolation (56, 57). Childhood adversity has also been associated with a greater burden of depression and anxiety in later life (58). Therefore, these findings suggest that the long-term association between childhood nutritional conditions and late-life cognition may be embedded within broader trajectories of educational, socioeconomic, and psychosocial advantage and disadvantage.

From a policy perspective, our findings highlight the potential value of a life-course approach that combines improvements in childhood nutrition with equitable access to education. Interventions aimed at reducing nutritional disadvantage early in life may help establish a stronger foundation for cognitive development, while greater educational opportunities may contribute to cognitive reserve and more favorable socioeconomic trajectories across adulthood. In later life, supportive social and community environments may further help maintain cognitive health by facilitating access to services, social participation, and aging in place (59, 60). Therefore, these considerations support integrated life-course strategies that address early nutritional disadvantage and educational inequality while maintaining supportive environments for healthy aging. More broadly, these findings may be viewed within a healthy-aging framework in which cognitive health reflects the accumulation and interaction of biological, educational, and social influences across the life course. Although not directly examined in our study, later-life social support and social inclusion may further shape wellbeing and cognitive health in older adulthood (61). Thus, the association between childhood nutritional conditions and late-life cognitive function may be embedded within broader life-course trajectories rather than reflecting a single biological pathway.

This study has several strengths, including a large community-based sample, assessment of multiple cognitive outcomes and domains, and examination of effect modification by parental literacy and potential mediation by adult education and other adulthood factors. However, several limitations should be considered. First, although childhood meat consumption, adult educational attainment, and late-life cognitive function represent chronologically ordered stages of the life course, the present study used a cross-sectional design and relied on retrospectively reported childhood exposure data, which may be subject to recall bias. Therefore, the observed associations should not be interpreted as evidence of causal relationships. The mediation analysis was specified according to this chronological ordering and may provide insights into potential life-course pathways; however, the observed indirect effects represent statistical associations within a chronologically ordered life-course framework rather than evidence of causal pathways. Longitudinal studies with prospectively assessed early-life exposures are needed to further establish temporal relationships and clarify potential causal mechanisms. Second, CMC is a crude proxy for childhood dietary and nutritional conditions. The questionnaire assessed the frequency but not the amount or type of meat consumed, total protein intake, other animal- or plant-based protein sources, energy intake, or overall dietary quality. CMC therefore should not be interpreted as a comprehensive measure of childhood nutritional status. Third, rresidual confounding by childhood socioeconomic circumstances is an important concern. In a historical setting characterized by famine and food scarcity, household financial resources and food access were likely important determinants of meat consumption. Parental literacy captures family educational resources but is not equivalent to childhood household economic status and may itself correlate with economic advantage. Because direct measures of childhood household income, wealth, or material conditions were unavailable, the observed associations with CMC and their variation by parental literacy may partly reflect unmeasured childhood socioeconomic circumstances rather than parental literacy *per se*. Historical evidence also suggests that animal-source foods were less widely available in China during the childhood years of many participants than in more recent decades (62). Therefore, our findings may be most applicable to populations that experienced similarly low levels of childhood meat availability and consumption and may not generalize directly to populations with substantially higher childhood meat intake. Finally, the study did not account for genetic factors, which may influence the development and progression of MCI. For example, APOE ε4 status has been identified as a significant predictor of cognitive transitions among individuals with MCI (63). APOE ε4 is also a major genetic risk factor for late-onset AD and has been associated with an increased risk of progression from MCI to AD dementia (64). Further studies to account for potential role and effect modification from genetic factors are warranted.

## 5 Conclusion

In conclusion, we found that more frequent CMC was associated with better late-life cognitive function across various domains (particularly orientation), and with lower odds of MCI. These associations were stronger among participants with literate parents, although this pattern may partly reflect broader childhood socioeconomic circumstances rather than parental literacy itself. Adult education showed the largest estimated indirect association, statistically accounting for a substantial proportion of the association between CMC and late-life cognitive function, a pattern consistent with a potential life-course pathway. These findings highlight the potential relevance of childhood nutritional and socioeconomic circumstances and subsequent educational attainment to cognitive health in later life, while longitudinal studies with prospectively assessed early-life exposures are needed to further evaluate these relationships and their potential causal mechanisms.

## Data Availability

The datasets presented in this article are not readily available because data that support findings are restricted to researchers who have permission from the Guangzhou Biobank Cohort Study, and so are not publicly available. Requests to access the datasets should be directed to Zhihao Chai, 1034879112@qq.com.

## References

[1] American Psychiatric Association. Diagnostic and Statistical Manual of Mental Disorders, Fifth Edition. Washington, DC: American Psychiatric Association (2013). Available online at: https://psychiatryonline.org/doi/book/10.1176/appi.books.9780890425596 (Accessed April 9, 2026).

[2] Bai W Chen P Cai H Zhang QG Su ZH Cheung T . Worldwide prevalence of mild cognitive impairment among community dwellers aged 50 years and older: a meta-analysis and systematic review of epidemiology studies. Age Ageing. (2022) 51:afac173. doi: 10.1093/ageing/afac173 35977150

[3] Jia LF Du YF Chu L Zhang ZJ Li FY Lyu DY . Prevalence, risk factors, and management of dementia and mild cognitive impairment in adults aged 60 years or older in China: a cross-sectional study. Lancet Public Health. (2020) 5:E661–71. doi: 10.1016/S2468-2667(20)30185-7 33271079

[4] Petersen RC. Mild cognitive impairment as a diagnostic entity. J Intern Med. (2004) 256:183–94. doi: 10.1111/j.1365-2796.2004.01388.x 15324362

[5] Digirolamo AM Ochaeta L Flores RMM. Early childhood nutrition and cognitive functioning in childhood and adolescence. Food Nutr Bull. (2020) 41:S31–40. doi: 10.1177/0379572120907763 32522121

[6] Kirolos A Harawa PP Chimowa T Divala O Freyne B Jones AG . Long-term outcomes after severe childhood malnutrition in adolescents in Malawi (LOSCM): a prospective observational cohort study. Lancet Child Adolesc. (2024) 8:280–9. doi: 10.1016/S2352-4642(23)00339-5 38368896

[7] Roberts M Tolar-Peterson T Reynolds A Wall C Reeder N Mendez GR. The effects of nutritional interventions on the cognitive development of preschool-age children: a systematic review. Nutrients. (2022) 14:532. doi: 10.3390/nu14030532 35276891 PMC8839299

[8] Zhang Z Gu D Hayward MD. Childhood nutritional deprivation and cognitive impairment among older Chinese people. Soc Sci Med. (2010) 71:941–9. doi: 10.1016/j.socscimed.2010.05.013 20591545 PMC4476240

[9] Rong HG Xi YD An Y Tao LW Zhang XN Yu HY . The correlation between early stages of life exposed to Chinese famine and cognitive decline in adulthood: nutrition of adulthood plays an important role in the link? Front Aging Neurosci. (2018) 9:444. doi: 10.3389/fnagi.2017.00444 29375368 PMC5767719

[10] Heys M Jiang CQ Schooling CM Zhang WS Cheng KK Lam TH . Is childhood meat eating associated with better later adulthood cognition in a developing population? Eur J Epidemiol. (2010) 25:507–16. doi: 10.1007/s10654-010-9466-0 20526800 PMC2903695

[11] Zhang Z Liu J Li L Xu H. The long arm of childhood in china: early-life conditions and cognitive function among middle-aged and older adults. J Aging Health. (2018) 30:1319–44. doi: 10.1177/0898264317715975 28645234

[12] Maurer J. Height, education and later-life cognition in Latin America and the Caribbean. Econ Hum Biol. (2010) 8:168–76. doi: 10.1016/j.ehb.2010.05.013 20678741

[13] Meng X Qian N Yared P. The institutional causes of China's Great Famine, 1959–1961. Rev Econ Stud. (2015) 82:1568–611. doi: 10.1093/restud/rdv016

[14] Dror DK Allen LH. The importance of milk and other animal-source foods for children in low-income countries. Food Nutr Bull. (2011) 32:227–43. doi: 10.1177/156482651103200307 22073797

[15] Bradley RH Corwyn RF. Socioeconomic status and child development. Annu Rev Psychol. (2002) 53:371–99. doi: 10.1146/annurev.psych.53.100901.135233 11752490

[16] Stern Y. Cognitive reserve in ageing and Alzheimer's disease. Lancet Neurol. (2012) 11:1006–12. doi: 10.1016/S1474-4422(12)70191-6 23079557 PMC3507991

[17] Shi LP Yao SH Wang W. Prevalence and distribution trends of mild cognitive impairment among Chinese older adults: a meta-analysis. Chin Gen Pract. (2022) 25:109–14. doi: 10.12114/j.issn.1007-9572.2021.00.315

[18] Jiang CQ Thomas GN Lam TH Schooling CM Zhang WS Lao XQ . Cohort profile: the guangzhou biobank cohort study, a Guangzhou-Hong Kong-Birmingham collaboration. Int J Epidemiol. (2006) 35:844–52. doi: 10.1093/ije/dyl131 16844769

[19] Jin YL Xu L Jiang CQ Zhang WS Pan J Zhu F . Association of hand grip strength with mild cognitive impairment in middle-aged and older people in guangzhou biobank cohort study. Int J Environ Res Public Health. (2022) 19:6464. doi: 10.3390/ijerph19116464 35682049 PMC9180839

[20] Tian YM Wang J Zhang WS Jiang CQ Li Jin Y Zhu T . Association of perceived stress with memory decline in older Chinese: the Guangzhou Biobank Cohort Study. J Affect Disord. (2023) 341:256–64. doi: 10.1016/j.jad.2023.08.122 37634823

[21] Wang J Li YR Jiang CQ Zhang WS Zhu T Zhu F . Chronotype and cognitive function: observational study and bidirectional Mendelian randomization. Eclinicalmedicine. (2022) 53:101713. doi: 10.1016/j.eclinm.2022.101713 36467458 PMC9716330

[22] Yeung SLA Jiang CQ Cheng KK Zhang WS Lam TH Leung GM . Genetically predicted 17beta-estradiol, cognitive function and depressive symptoms in women: a Mendelian randomization in the Guangzhou Biobank Cohort Study. Prev Med. (2016) 88:80–5. doi: 10.1016/j.ypmed.2016.03.002 27036929

[23] Xu L Jiang CQ Lam TH Sen Zhang W Cherny SS Thomas GN . Sleep duration and memory in the elderly Chinese: longitudinal analysis of the Guangzhou Biobank cohort study. Sleep. (2014) 37:1736–45. doi: 10.5665/sleep.4162 25364069 PMC4196057

[24] Deng HB Macfarlane DJ Thomas GN Lao XQ Jiang CQ Cheng KK . Reliability and validity of the IPAQ-Chinese: the Guangzhou Biobank Cohort Study. Med Sci Sport Exer. (2008) 40:303–7. doi: 10.1249/mss.0b013e31815b0db5 18202571

[25] Huang YY Jiang CQ Xu L Zhang WS Zhu F Jin YL . Mortality in relation to changes in physical activity in middle-aged to older Chinese: an 8-year follow-up of the Guangzhou Biobank Cohort Study. J Sport Health Sci. (2021) 10:430–8. doi: 10.1016/j.jshs.2020.08.007 32827710 PMC8343063

[26] Chan AC. Clinical validation of the Geriatric Depression Scale (GDS): Chinese version. J Aging Health. (1996) 8:238–53. doi: 10.1177/089826439600800205 10160560

[27] Lin QH Jiang CQ Lam TH Xu L Jin YL Cheng KK. Past occupational dust exposure, depressive symptoms and anxiety in retired Chinese factory workers: the Guangzhou Biobank Cohort Study. J Occup Health. (2014) 56:444–52. doi: 10.1539/joh.14-0100-OA 25214191

[28] Ng TP Niti M Fones C Yap KB Tan WC. Co-morbid association of depression and COPD: A population-based study. Respir Med. (2009) 103:895–901. doi: 10.1016/j.rmed.2008.12.010 19136238

[29] Jin Y Liang J Hong C Liang R Luo Y. Cardiometabolic multimorbidity, lifestyle behaviours, and cognitive function: a multicohort study. Lancet Healthy Longev. (2023) 4:e265–73. doi: 10.1016/S2666-7568(23)00054-5 37150183

[30] Li Y Li Y Gu X Liu Y Dong D Kang JH. Long-term intake of red meat in relation to dementia risk and cognitive function in US adults. Neurology. (2025) 104:e210286. doi: 10.1212/WNL.0000000000210286 39813632 PMC11735148

[31] Liang WY Ye XM Li T. How does parental involvement affect the cognitive ability of migrant children: an empirical study based on CEPS baseline data. J Educ Stud. (2018) 14:80–94. doi: 10.14082/j.cnki.1673-1298.2018.01.011

[32] Liu J Cheng M Wu C. Parental education level and child cognitive ability: an analysis based on evidence from the China education panel survey. Best Evid Chin Educ. (2025) 19:1923–6. doi: 10.15354/bece.25.ar141

[33] Stein AD Adair LS Donati G Wray C Richter LM Norris SA . Early-life stature, preschool cognitive development, schooling attainment, and cognitive functioning in adulthood: a prospective study in four birth cohorts. Lancet Glob Health. (2023) 11:E95–E104. doi: 10.1016/S2214-109X(22)00448-X 36521958 PMC9764453

[34] Ahmed A Kramer MS Bernard JY Perez Trejo ME Martin RM Oken E . Early childhood growth trajectory and later cognitive ability: evidence from a large prospective birth cohort of healthy term-born children. Int J Epidemiol. (2020) 49:1998–2009. doi: 10.1093/ije/dyaa105 32743654 PMC8208486

[35] Heys M Schooling CM Jiang C Adab P Cheng KK Lam TH . Childhood growth and adulthood cognition in a rapidly developing population. Epidemiology. (2009) 20:91–9. doi: 10.1097/EDE.0b013e3181880396 19244658

[36] Huang TL Carlson MC Fitzpatrick AL Kuller LH Fried LP Zandi PP. Knee height and arm span - a reflection of early life environment and risk of dementia. Neurology. (2008) 70:1818–26. doi: 10.1212/01.wnl.0000311444.20490.98 18458216

[37] Zhang Z Gu D Hayward MD. Early life influences on cognitive impairment among oldest old Chinese. J Gerontol B Psychol Sci Soc Sci. (2008) 63:S25–33. doi: 10.1093/geronb/63.1.S25 18332198

[38] Zhang ZX Plassman BL Xu Q Zahner GEP Wu B Gai MY . Lifespan influences on mid- to late-life cognitive function in a Chinese birth cohort. Neurology. (2009) 73:186–94. doi: 10.1212/WNL.0b013e3181ae7c90 19620606 PMC2843580

[39] Zheng H Cagney K Choi Y. Predictors of cognitive functioning trajectories among older Americans: a new investigation covering 20 years of age- and non-age-related cognitive change. PLoS ONE. (2023) 18:e0281139. doi: 10.1371/journal.pone.0281139 36753483 PMC9907834

[40] Jeong SK Kim JM Kweon SS Shin MH Seo MW Kim YH. Does arm length indicate cognitive and functional reserve? Int J Geriatr Psychiatry. (2005) 20:406–12. doi: 10.1002/gps.1295 15852453

[41] Kim JM Stewart R Shin S Kim SW Yang SJ Yoon JS. Associations between head circumference, leg length and dementia in a Korean population. Int J Geriatr Psych. (2008) 23:41–8. doi: 10.1002/gps.1833 17535018

[42] Kim JM Stewart R Shin IS Yoon JS. Limb length and dementia in an older Korean population. J Neurol Neurosurg Psychiatry. (2003) 74:427–32. doi: 10.1136/jnnp.74.4.427 12640055 PMC1738375

[43] Ding RX He P. Associations between childhood adversities and late-life cognitive function: Potential mechanisms. Soc Sci Med. (2021) 291:114478. doi: 10.1016/j.socscimed.2021.114478 34649168

[44] Lin ZE Chen X. Adverse childhood circumstances and cognitive function in middle-aged and older Chinese adults: lower level or faster decline? Ssm-Popul Hlth. (2021) 14:100767. doi: 10.1016/j.ssmph.2021.100767 33855158 PMC8025052

[45] Lyu J Burr JA. Socioeconomic status across the life course and cognitive function among older adults: an examination of the latency, pathways, and accumulation hypotheses. J Aging Health. (2016) 28:40–67. doi: 10.1177/0898264315585504 26006338

[46] Ritchie K Jaussent I Stewart R Dupuy AM Courtet P Malafosse A . Adverse childhood environment and late-life cognitive functioning. Int J Geriatr Psych. (2011) 26:503–10. doi: 10.1002/gps.2553 21445999

[47] Fernstrom JD. Dietary amino acids and brain function. J Am Diet Assoc. (1994) 94:71–7. doi: 10.1016/0002-8223(94)92045-1 7903674

[48] Zeisel SH Da Costa KA. Choline: an essential nutrient for public health. Nutr Rev. (2009) 67:615–23. doi: 10.1111/j.1753-4887.2009.00246.x 19906248 PMC2782876

[49] Lozoff B Georgieff MK. Iron deficiency and brain development. Semin Pediatr Neurol. (2006) 13:158–65. doi: 10.1016/j.spen.2006.08.004 17101454

[50] Watanabe F. Vitamin B12 sources and bioavailability. Zhonghua Yu Fang Yi Xue Za Zhi. (2007) 232:1266–74. doi: 10.3181/0703-MR-67 17959839

[51] Kong XY Ren RN Liu LJ Sheng XL Bai JS. Effects of dietary zinc deficiency on brain development, learning, and memory in young rats. Chin J Prev Med. (1997) 31:295–8. 9812610

[52] Noble KG Grieve SM Korgaonkar MS Engelhardt LE Griffith EY Williams LM . Hippocampal volume varies with educational attainment across the life-span. Front Hum Neurosci. (2012) 6:307. doi: 10.3389/fnhum.2012.00307 23162453 PMC3494123

[53] Langa KM. Is the risk of Alzheimer's disease and dementia declining? Alzheimers Res Ther. (2015) 7:34. doi: 10.1186/s13195-015-0118-1 25815064 PMC4374373

[54] Dannefer D. Cumulative advantage/disadvantage and the life course: cross-fertilizing age and social science theory. J Gerontol B Psychol Sci Soc Sci. (2003) 58:S327–37. doi: 10.1093/geronb/58.6.S327 14614120

[55] Hatch SL. Conceptualizing and identifying cumulative adversity and protective resources: implications for understanding health inequalities. J Gerontol B Psychol Sci Soc Sci 60 Spec No 2. (2005) 60:130–4. doi: 10.1093/geronb/60.Special_Issue_2.S130 16251584

[56] Zhu Y Yin H Zhong X Wang L Tang X Zhang Q . Adverse childhood experiences impair cognitive function via social isolation and functional limitations in Chinese middle-aged and older adults. Sci Rep. (2025) 15:27999. doi: 10.1038/s41598-025-12822-w 40745458 PMC12314117

[57] Shi S Kou W Bian Z Chen X Song L Fu L . The impact of adverse childhood experiences on cognitive function among middle-aged and older Chinese adults: Multiple mediators of cognitive reserve and depressive symptoms. J Affect Disord. (2025) 368:258–65. doi: 10.1016/j.jad.2024.09.049 39278468

[58] Lian J Kiely KM Callaghan BL Anstey KJ. Childhood adversity is associated with anxiety and depression in older adults: a cumulative risk and latent class analysis. J Affect Disord. (2024) 354:181–90. doi: 10.1016/j.jad.2024.03.016 38484890

[59] Bayar R Cebi Karaaslan K. The geography of happiness in old age: insights from older adults in Turkiye. Urban Stud. (2026). doi: 10.1177/00420980261443182. [Epub ahead of print].

[60] Bayar R Cebi Karaaslan K. From data to action: shaping age-friendly urban policies based on older adults' satisfaction. Soc Sci J. (2026). doi: 10.1080/03623319.2026.2680561 [Epub ahead of print].

[61] Cebi Karaaslan K Bayar R Ozturk A. Life satisfaction in later life: the role of health, social support, and social exclusion in Turkiye. BMC Geriatr. (2026) 26:706. doi: 10.1186/s12877-026-07438-2 41957742 PMC13191974

[62] Wang ZH Zhang B Wang HJ Zhang JG Du WW Su C. Trend in meat consumption patterns among Chinese children in nine provinces (autonomous regions) aged 6 to 17 years between 1991 and 2011. Zhonghua Yu Fang Yi Xue Za Zhi. (2013) 47:826–31. 24351564

[63] Qin Y Han HJ Li Y Cui J Jia HX Ge XY . Estimating bidirectional transitions and identifying predictors of mild cognitive impairment. Neurology. (2023) 100:E297–307. doi: 10.1212/WNL.0000000000201386 36220593 PMC9869761

[64] Troutwine BR Hamid L Lysaker CR Strope TA Wilkins HM. Apolipoprotein E and Alzheimer's disease. Acta Pharm Sin B. (2022) 12:496–510. doi: 10.1016/j.apsb.2021.10.002 35256931 PMC8897057

[65] Folstein MF Folstein SE McHugh PR. “Mini-mental state”. A practical method for grading the cognitive state of patients for the clinician. J Psychiatr Res. (1975) 12:189–98. doi: 10.1016/0022-3956(75)90026-6 1202204

