# Supplementary material for: Association of childhood meat consumption with late-life cognitive function: mediation by adult education and moderation by parental literacy
Source: Front Public Health. 2026 Oct 5;14:1878281. doi: 10.3389/fpubh.2026.1878281 (PMC13677485; doi:10.3389/fpubh.2026.1878281)
Supplement: Supplementary file 1 [file Supplementary_file_1.pdf]

Supplementary Table 1 Diagnostic assessment of the multiple linear regression model

Diagnostic item	Assessment method or criterion	Result
Residual normality	Histogram and normal P–P plot of standardized residuals	Some departure from normality, particularly in the lower tail
Homoscedasticity	Standardized residuals versus standardized predicted values plot	Some evidence of heteroscedasticity
Potential outliers	Absolute studentized deleted residual greater than 3	131 of 8,865 participants (1.5%)
High-leverage observations	Leverage value greater than $3p/n$	59 of 8,865 participants (0.7%)
Influential observations	Cook's distance greater than 1	0 participants
Maximum Cook's distance	-	0.012
Maximum leverage value	-	0.014
Multicollinearity	Variance inflation factor (VIF)	1.038–5.132
Multicollinearity	Tolerance	0.195–0.964

p represents the number of estimated model parameters, including the intercept, and *n* represents the analytical sample size.

Supplementary Table 2 Sensitivity analyses of the association between childhood meat consumption and MMSE score

**Childhood meat consumption**	**Primary analysis, *n*=8,865, β (95% *CI*)**	**Excluding potential outliers, *n*=8,734, β (95% *CI*)**	**Robust standard-error analysis, *n*=8,865, β (95% *CI*)**
Never or about once a year	Ref (0.00)	Ref (0.00)	Ref (0.00)
About once a month	0.14 (-0.01-0.29)	0.10 (-0.03-0.02)	0.15 (-0.03-0.32)
About once a week	0.33 (0.18-0.48)***	0.27 (0.15-0.40)***	0.36 (0.20-0.52)***
Almost daily	0.52 (0.33-0.71)***	0.44 (0.28-0.60)***	0.50 (0.32-0.68)***

All models were adjusted for sex, age, parental literacy, adult education, family annual income, occupation, marital status, physical activity, BMI, smoking status, drinking status, self-rated health, cardiovascular disease, diabetes and depressive symptoms.

Potential outliers were defined as observations with an absolute studentized deleted residual greater than 3. Robust standard errors were estimated using a generalized linear model with a normal distribution, an identity link function, and a robust sandwich covariance estimator.

MMSE, Mini-Mental State Examination; β, unstandardized regression coefficient; *CI*, confidence interval.

***P <0.001.

Supplementary Figure 1 Assessment of residual normality in the fully adjusted multiple linear regression model

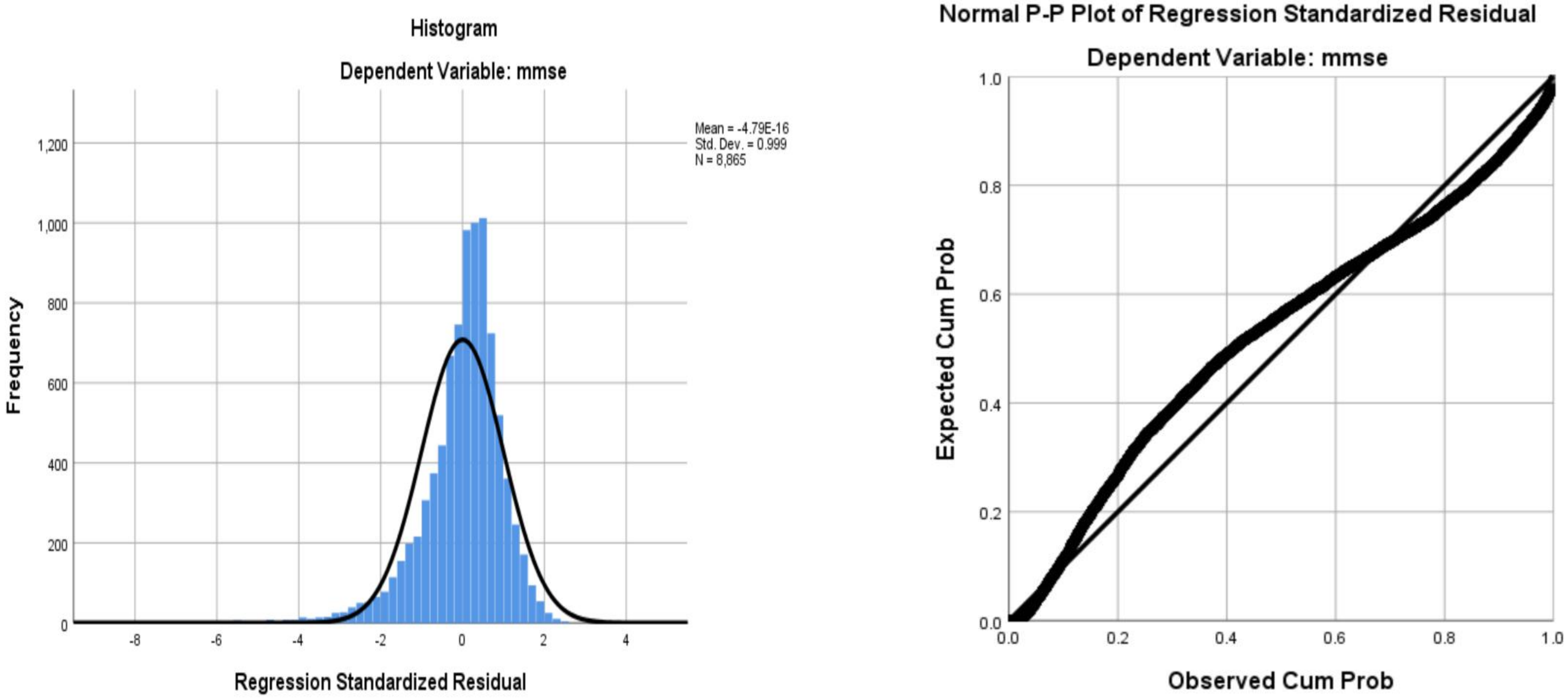

Panel A: Histogram of standardized residuals

Panel B: Normal P–P plot of standardized residuals

(A) Histogram of regression standardized residuals with a superimposed normal distribution curve, which is used to intuitively reflect the distribution pattern of residuals.

(B) Normal P–P plot of regression standardized residuals, which helps to verify whether residuals follow a normal distribution.

The plots were used to visually assess the normality of the residual distribution in the fully adjusted multiple linear regression model. As we know, the standardized residual approximately follows the standard normal distribution, and the probability that the standardized residual of an experimental point falls outside the interval (-2,2) is ≤0.05. If a certain experimental point's standardized residual is outside this interval, it can be judged as an abnormal experimental point with 95% confidence and not participate in the regression line fitting. Some deviation from the expected normal distribution was observed. The analysis included 8,865 participants.

Supplementary Figure S2 Scatterplot of standardized residuals against standardized predicted values in the fully adjusted multiple linear regression model

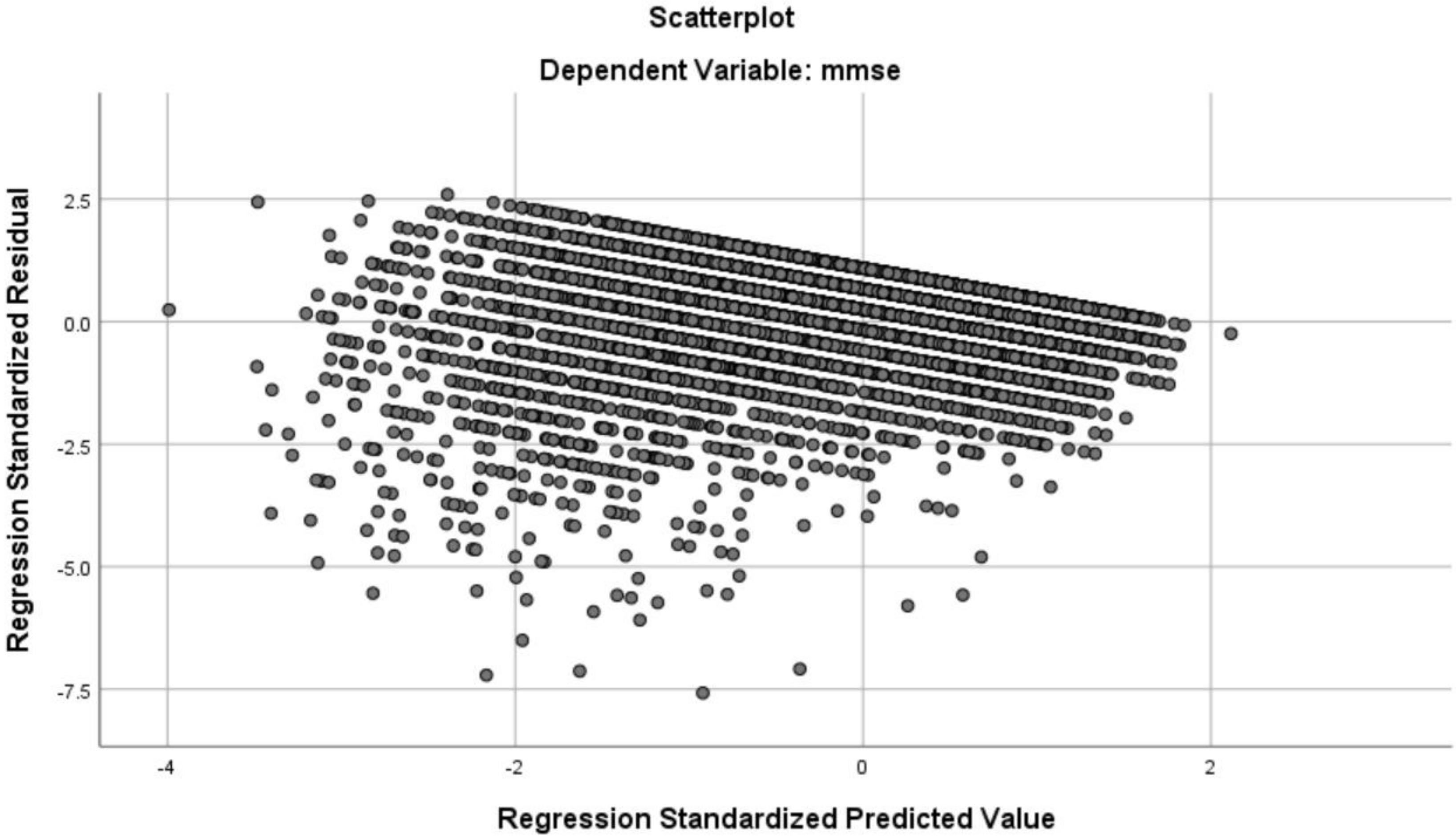

Regression standardized residuals were plotted against regression standardized predicted values to visually assess the homoscedasticity assumption. As observed in the plot, the variation in residual dispersion across the range of predicted values showed a pattern where the degree of residual variation changed with the level of predicted values, which indicates a violation of the homoscedasticity assumption. The analysis included 8,865 participants.
